# Interpretable machine learning for identifying determinants of high hypertension burden under extreme heat vulnerability: evidence from Maryland, USA

**DOI:** 10.3389/fpubh.2026.1894531

**Published:** 2026-07-15

**Authors:** Binbin Peng

**Affiliations:** School of Government, Nanjing University, Nanjing, China

**Keywords:** emergency management, extreme heat, health risk, hypertension, machine learning, spatial heterogeneity, health equity

## Abstract

Extreme heat poses increasing risks to cardiovascular health, yet fine-scale determinants of heat-related hypertension burden remain insufficiently understood. This study examines high hypertension burden in a heat-vulnerability context across 1,385 census tracts in Maryland using an interpretable machine learning framework. An XGBoost model was developed to classify census tracts with high hypertension burden using demographic, socioeconomic, built-environment, heat anomaly, and adaptive-capacity variables. SHapley Additive exPlanations (SHAP) were then used to identify key predictors, assess their contribution to model predictions, and examine non-linear effects. The model achieved strong predictive performance, with an accuracy of 0.819, balanced accuracy of 0.806, ROC-AUC of 0.821, and PR-AUC of 0.909. Results show substantial spatial heterogeneity in hypertension burden, with high-burden tracts concentrated in Baltimore City, Prince George's County, southern Maryland, western Maryland, and parts of the Eastern Shore. SHAP results indicate that African-American population share, older-adult population share, and low educational attainment were the strongest predictors, followed by summer maximum air temperature, non-vegetated land area, and lack of air conditioning. Dependence plots further reveal non-linear and threshold-like relationships, suggesting that predicted risk increases sharply beyond certain levels of demographic vulnerability, educational disadvantage, heat exposure, and limited cooling access. These findings indicate that high hypertension burden in a heat-vulnerability context is shaped by the intersection of structural social vulnerability, demographic susceptibility, environmental exposure, and household adaptive capacity. The study demonstrates the value of combining XGBoost and SHAP for tract-level heat-health risk assessment and provides policy-relevant evidence for targeted heat adaptation, cooling assistance, and public health preparedness in Maryland.

## 1 Introduction

Extreme heat is increasingly recognized as a major climate-sensitive threat to public health. A growing body of evidence indicates that anthropogenic climate change has intensified the health burden associated with heat exposure, including excess morbidity, mortality, and other measurable health losses (1). Heatwaves are projected to become more frequent, more intense, and longer lasting, particularly in urbanized regions where regional warming interacts with the built environment to produce persistent local heat conditions (2–6). Extreme heat can increase cardiovascular strain, impair thermoregulation, elevate dehydration risk, and aggravate pre-existing cardiometabolic conditions, thereby linking climate-related environmental exposure to chronic disease burden and population health vulnerability, and meanwhile, broader evidence reinforces the need to understand extreme heat as a compound urban risk that affects public health, mobility safety, and local adaptation planning (7, 8).

Hypertension is a particularly important outcome in this context. As a highly prevalent cardiovascular condition, hypertension can increase physiological sensitivity to thermal stress and may contribute to broader heat-related cardiovascular morbidity. Prior studies have established associations between high temperature and adverse health outcomes, including mortality, heat-related illness, and cardiovascular hospitalizations (9–11). However, much of the existing heat-health literature has focused on mortality, emergency department visits, or broad vulnerability indices, while fine-scale spatial variation in hypertension burden under heat-health vulnerability contexts has received less empirical attention. This gap is consequential because hypertension burden is not shaped by temperature exposure alone. It reflects the co-occurrence of environmental heat, socioeconomic disadvantage, demographic susceptibility, land-cover conditions, and household-level adaptive capacity, all of which are central concerns for environmental health equity and public health preparedness.

Neighborhood conditions are central to this process. Older adults, socially isolated residents, and individuals with chronic health conditions are frequently identified as heat-sensitive populations (12–15). Socioeconomic disadvantage may further increase risk by limiting access to preventive care, stable housing, cooling resources, and other protective infrastructure (16–19). At the same time, land-cover and built-environment characteristics influence the intensity and duration of local heat exposure. Impervious surfaces, reduced vegetation, limited tree canopy, and urban heat island effects can amplify thermal stress at the neighborhood scale (20–23). Recent evidence also shows that exposure to urban heat island intensity is unequally distributed across racial and socioeconomic groups in major U.S. cities, reinforcing the need to examine heat-health risk through a spatial equity lens (24).

These intersecting pathways suggest that hypertension burden under heat-health vulnerability should be analyzed as a spatially structured and socially patterned public health outcome. Peng et al. argue that heat-health risk cannot be adequately represented by a single dimension or by the conventional separation of hazard, exposure, and vulnerability; instead, it reflects multiple socio-environmental and socio-spatial processes that vary across census tracts (25). Their Maryland-based analysis provides an important foundation for understanding heat-health risk as a complex spatial phenomenon, but it does not directly model tract-level hypertension burden as a specific public health outcome. Building on this line of work, the present study shifts the analytical focus from classifying general heat-health risk dimensions to explaining the social and environmental predictors of high hypertension prevalence through an interpretable prediction framework.

A second limitation in prior research concerns methodological form. Many heat vulnerability assessments rely on composite indices that aggregate demographic, socioeconomic, environmental, and health-related indicators into a single score (21, 26–29). Such indices are useful for screening and communication, but they may obscure the marginal influence of individual predictors, depend on weighting or dimensionality-reduction decisions, and provide limited insight into non-linear exposure-response patterns. Linear statistical models, while valuable for estimating average associations, may also be insufficient when predictor effects vary across thresholds or depend on interactions among environmental exposure, social vulnerability, baseline health, and adaptive capacity. These limitations are especially relevant for public health risk identification, where the influence of heat exposure, neighborhood conditions, and social vulnerability on hypertension burden may intensify only after certain exposure or vulnerability levels are reached.

Adaptive capacity is another important but unevenly incorporated dimension of heat-health research. Air-conditioning access can reduce indoor heat exposure and has been associated with lower heat-related morbidity and mortality in prior studies (12, 30, 31). Yet cooling access is often absent from spatial vulnerability assessments or treated as a secondary contextual factor rather than as a central predictor. This omission may lead to incomplete estimates of neighborhood-level public health vulnerability because areas with similar outdoor heat exposure may differ substantially in residents' ability to reduce personal exposure indoors. Related research also suggests that heat can affect health through behavioral and social pathways, including changes in diet and consumption patterns that may disproportionately affect disadvantaged groups (32). Although these behavioral mechanisms are not directly modeled here, they further support the premise that heat-related health burdens are mediated by unequal resources and adaptive opportunities.

To address these gaps, this study examines high hypertension prevalence across 1,385 census tracts within a heat-health vulnerability framework using an interpretable machine learning approach. The analysis applies an Extreme Gradient Boosting model (XGBoost) to predict tract-level high hypertension burden in a heat-vulnerability context from environmental exposure, land-cover characteristics, demographic susceptibility, socioeconomic vulnerability, baseline health conditions, and air-conditioning access (33). SHapley Additive exPlanations (SHAP) are then used to interpret model predictions by estimating the contribution of each predictor and by revealing how influential variables affect predicted risk across their observed ranges (34). This framework is designed not only to improve predictive performance, but also to identify which socio-environmental predictors matter most, how different vulnerability domains jointly shape spatial disparities in hypertension burden, and whether key variables exhibit non-linear or threshold effects relevant to public health intervention.

By centering high hypertension prevalence as the health outcome and census tracts as the unit of analysis, this study contributes to environmental public health and heat-health vulnerability research in three ways. First, it extends existing heat vulnerability research by examining a specific chronic cardiovascular burden rather than relying solely on general vulnerability indices or mortality-based measures. Second, it advances a flexible and interpretable modeling strategy capable of detecting non-linear and interaction-dependent relationships among environmental exposure, land-cover conditions, demographic susceptibility, socioeconomic vulnerability, and adaptive capacity. Third, it provides evidence for spatially targeted public health action, including heat-health surveillance, cooling-resource allocation, chronic disease prevention, urban greening, and neighborhood-scale heat adaptation planning.

## 2 Literature review

### 2.1 Heat exposure, built environment, and cardiovascular health risk

Extreme heat is a well-established environmental hazard associated with increased mortality and morbidity, particularly during prolonged heatwave events. Epidemiological studies have shown that high temperature and heatwaves are associated with elevated cardiovascular risk, including cardiovascular mortality, hospitalizations, and other acute cardiovascular outcomes (9, 11, 35, 36). This evidence is especially relevant to hypertension because heat exposure can increase cardiovascular workload, disrupt thermoregulation, and intensify physiological stress among populations with pre-existing cardiovascular conditions.

Heat exposure is not determined by ambient air temperature alone. Land surface temperature, humidity, urban heat island intensity, surface materials, and neighborhood land cover all shape the degree to which residents experience thermal stress (22, 26, 37, 38). Impervious surfaces, dense development, and limited vegetation can intensify local heat accumulation, whereas tree canopy and green space can reduce heat exposure through shading and evapotranspiration (20–23). Recent work further shows that urban heat island intensity and heat persistence jointly contribute to modeled urban heat events, suggesting that local heat exposure reflects both the magnitude and duration of elevated temperature conditions (5). In addition, heat exposure is spatially unequal: across major U.S. cities, people of color and residents living in poverty are disproportionately exposed to higher surface urban heat island intensity (24). These findings support the need for fine-scale analysis of high hypertension burden in a heat-vulnerability context at the census-tract level, where environmental exposure and social inequality intersect.

### 2.2 Social vulnerability, baseline health susceptibility, and adaptive capacity

Heat-health risk is unevenly distributed across populations because exposure interacts with social vulnerability, health susceptibility, and adaptive capacity. Social vulnerability frameworks emphasize that communities differ in their ability to prepare for, cope with, and recover from environmental hazards (16, 17). In heat-health research, older adults, low-income households, racial and ethnic minority populations, socially isolated individuals, residents with limited educational attainment, and people with constrained access to health care are frequently identified as heat-vulnerable groups (21, 29, 39). These social conditions may increase risk by limiting access to cooling, transportation, health services, stable housing, and other protective resources.

Baseline health conditions are also central to heat susceptibility. Chronic diseases such as hypertension, coronary heart disease, asthma, chronic obstructive pulmonary disease, diabetes, chronic kidney disease, and depression can increase physiological sensitivity to heat and may amplify the health consequences of extreme temperature exposure (11, 35, 36).

Adaptive capacity further modifies heat-health risk. Air conditioning is one of the most direct household-level protections against indoor heat exposure, but access to cooling is shaped by income, housing quality, energy affordability, and infrastructure. Studies have shown that lack of air conditioning and poor pre-existing health status are associated with greater heat-related illness risk, while air-conditioned environments can reduce heat-related mortality and morbidity (12, 30, 40). Therefore, air-conditioning access should not be treated merely as a household amenity; it is also an adaptive-capacity indicator that reflects broader inequalities in the ability to avoid heat exposure. By integrating demographic vulnerability, chronic disease burden, and cooling access, high hypertension burden in a heat-vulnerability context can be conceptualized as a compound outcome shaped by both exposure and unequal capacity to adapt. These findings suggest that heat-health vulnerability is inherently spatial and multidimensional. Because exposure, susceptibility, and adaptive capacity vary substantially across neighborhoods and census tracts, a spatially explicit assessment is needed to identify where hypertension burden may overlap with heat-related vulnerability (41).

### 2.3 Interpretable machine learning for spatial heat-health risk prediction

Traditional heat vulnerability assessments often rely on composite indices that combine environmental, demographic, socioeconomic, and health-related indicators into a single score (21, 26, 29). These approaches are useful for visualization and planning, but they may obscure the relative contribution of individual predictors, depend on weighting assumptions, and provide limited insight into non-linear or threshold effects. Peng et al. highlight that heat-health risk is multidimensional and spatially heterogeneous, and that single-index approaches may be insufficient for representing complex socio-environmental risk structures (25). This limitation is particularly important for hypertension-focused heat-health analysis, where risk may emerge from non-linear interactions among heat exposure, baseline health conditions, social vulnerability, land-cover characteristics, and adaptive capacity.

Machine learning provides a flexible alternative for modeling such complexity. Tree-based ensemble models, including random forest and extreme gradient boosting, can capture non-linear effects and higher-order interactions without imposing a strictly linear functional form (33). In recent heat-health studies, machine learning has been used to estimate spatially detailed heat-related mortality and morbidity risk and to identify the relative importance of climatic, demographic, socioeconomic, and built-environment predictors (42, 43). However, predictive performance alone is insufficient for public health and environmental justice applications because policy relevance requires interpretable evidence on why risk is elevated and which factors drive spatial disparities. SHAP address this interpretability challenge by decomposing model predictions into feature-level contributions (34). In heat-risk research, SHAP can identify globally important predictors, show whether their effects are positive or negative, and reveal non-linear or threshold patterns across observed predictor ranges (42, 43).

## 3 Materials and methods

### 3.1 Study area

**Figure 1:**
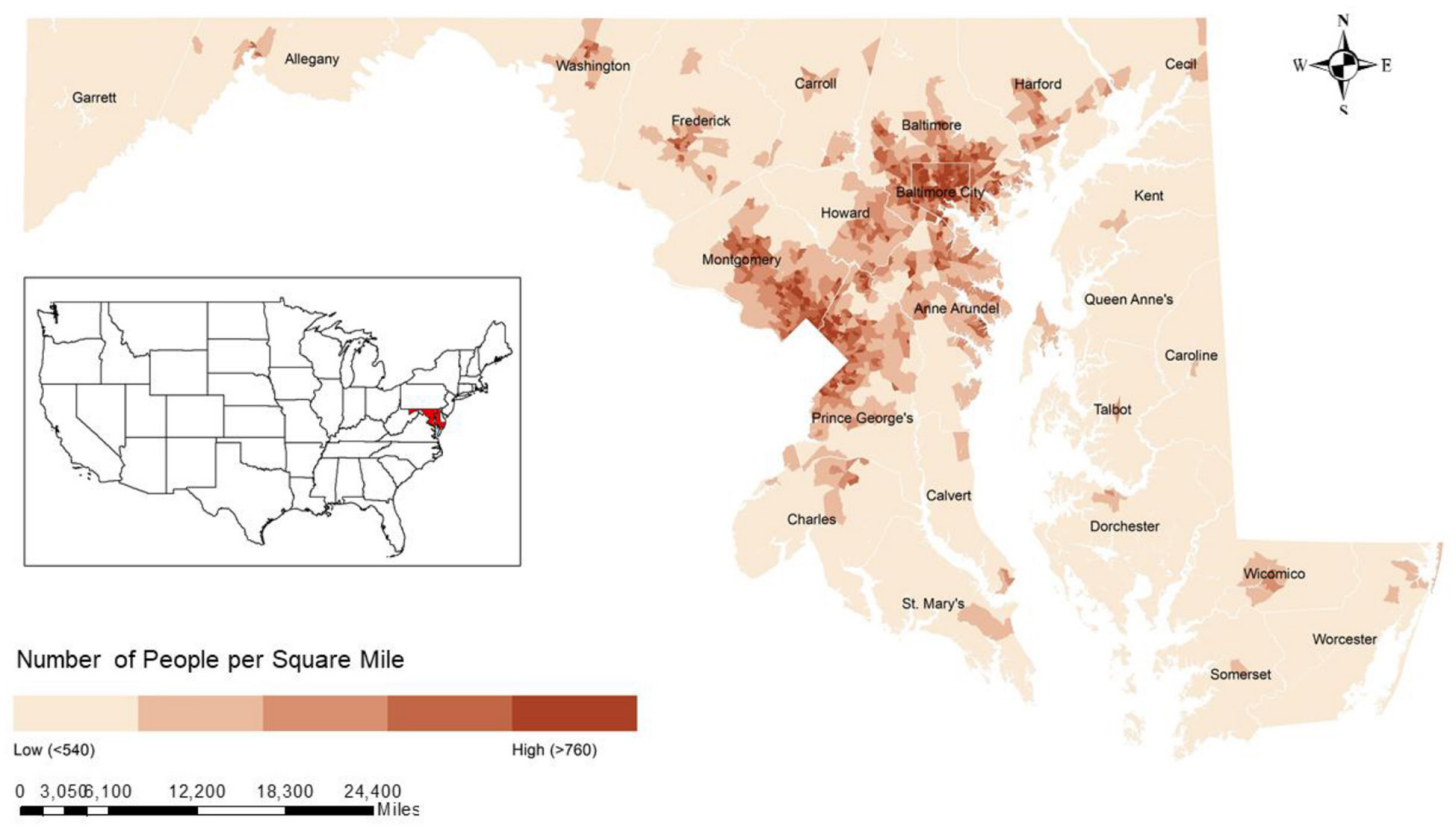
Study area and population density at the census tract level.

Maryland is located in the Mid-Atlantic region of the United States, bordering the District of Columbia, Virginia, West Virginia, Pennsylvania, and Delaware, as shown in Figure 1. The state contains a diverse mixture of densely urbanized areas, suburban communities, coastal zones, and rural landscapes. Maryland's population is unevenly distributed across census tracts, with the highest densities concentrated in the Baltimore metropolitan area and the Washington, D.C. suburban corridor, particularly in Montgomery and Prince George's Counties, while western Maryland and much of the Eastern Shore remain comparatively low-density. In recent years, extreme heat has become a persistent climate-related public health concern for Maryland. Previous state-level evidence indicates that summertime extreme heat events in Maryland have increased substantially in recent decades and are associated with multiple heat-sensitive health outcomes, including cardiovascular and respiratory conditions (44). Recent heat-health research using Maryland as a case study also highlights that heat-related health risk varies considerably across census tracts due to differences in social vulnerability, land-cover conditions, heat exposure, and adaptive capacity (41). Therefore, Maryland provides an appropriate empirical context for investigating how environmental, socioeconomic, demographic, and household-level factors jointly shape tract-level high hypertension burden in a heat-vulnerability context.

### 3.2 Data and methods

This study integrated multi-source census-tract-level data to characterize high hypertension burden in a heat-vulnerability context across Maryland, as shown in Table 1. Demographic variables were obtained from the U.S. Census and linked to census-tract geometries using TIGER/Line boundary files (45). Socioeconomic vulnerability variables were derived mainly from the American Community Survey (46), and adaptive capacity variable, i.e. household air-conditioning information was obtained from ZTRAX (47). Built environmental conditions were calculated from the national land cover database (48). Heat anomaly variables were derived from USGS Landsat 8 and NOAA weather-station records (49). The dependent health outcome was obtained from CDC PLACES, which provides model-based small-area estimates of chronic disease indicators at the census-tract level. All variables were harmonized to the census-tract scale to support spatially consistent modeling and interpretation.

**Table 1:** Data sources and variable definitions.

Dimension	Source	Variable	Definition
Demographic characteristics	US census, TIGER	Pop65	Percent population aged over 65
		Black	Percent population as African-American only
		Hispanic	Percent population as Hispanic
Socioeconomic vulnerability	American Community Survey	PopUnderPoverty	Percent population below the poverty line
		GiniIndex	Gini index of income inequity
		NoHealthInsurance	Percent population with no health insurance
		LessHS	Percent population with less than a high school diploma
		PopLivingAlone	Percent population living alone
		HouseholderOver65LivingAlone	Percent householder over 65 living alone
Adaptive capacity	ZTRAX	PctNoAC	Percent households with no air conditioning
Built Environmental condition	MRLC	NonVegeRatio	Percent census-tract area not covered in vegetation
		ImpSurfaceRatio	Percent census-tract area with impervious surfaces
		TreeCanopyRatio	Percent census-tract area with tree canopy
Heat anomaly	USGS Landsat 8, NOAA	LSTmax	Land surface heat anomaly in summer
		AirTmax	Air temperature anomaly in summer
Health outcome	CDC PLACES	BPHIGH	Percent population with high blood pressure
		BPHIGH_high	Indicator of whether a census tract has high hypertension burden

#### 3.2.1 Dependent variable

The original health outcome was tract-level hypertension prevalence derived from CDC PLACES. This continuous variable, hereafter referred to as BPHIGH, represents the estimated percentage of adults aged 18 years and older who reported having been told by a health professional that they had high blood pressure (50). Because the machine-learning model was designed as a binary classification model, BPHIGH was converted into a high-burden indicator, BPHIGH_high. Census tracts with BPHIGH greater than or equal to the sample median were coded as 1, indicating high hypertension burden, while tracts below the median were coded as 0, indicating lower hypertension burden. The median threshold was used because no universally accepted census-tract-level public-health cutoff exists for defining high hypertension burden, and this rule produced a nearly balanced classification for model training and evaluation.

#### 3.2.2 Independent and control variables

Heat anomaly variables were included to capture direct thermal exposure. Land surface heat anomaly in summer (LSTmax) was derived from USGS Landsat 8 data and represents spatial variation in surface thermal conditions. Air temperature anomaly in summer (AirTmax) was derived from NOAA weather-station data and represents variation in ambient summer heat exposure. Together, these two variables distinguish surface heat conditions from near-surface air temperature exposure, allowing the model to account for both land-cover-driven thermal patterns and broader atmospheric heat conditions.

The control variables were selected to represent environmental exposure, social-economic vulnerability, demographic susceptibility, built-environment conditions, and adaptive capacity. Demographic characteristics included the percentage of the population aged over 65 years (Pop65), the percentage of the population identified as African-American only (Black), and the percentage of the population identified as Hispanic (Hispanic). These variables capture population groups that may experience differential heat-health vulnerability due to age-related physiological sensitivity, structural inequality, and uneven access to protective resources.

Socioeconomic characteristics included the percentage of the population below the poverty line (PopUnderPoverty), the Gini index of income inequality (GiniIndex), the percentage of the population without health insurance (NoHealthInsurance), and the percentage of the population with less than a high school diploma (LessHS). These variables reflect economic disadvantage, inequality, health-care access, and educational attainment, all of which may influence the ability of residents to prepare for, respond to, and recover from extreme heat exposure. Additional socioeconomic and household-context indicators included the percentage of the population living alone (PopLivingAlone) and the percentage of householders aged over 65 living alone (HouseholderOver65LivingAlone). In addition, the percentage of households without air conditioning (PctNoAC) was used as a direct indicator of household-level adaptive capacity, while living-alone variables captured potential social isolation during heat events.

Built-environment variables described land-cover conditions that may affect local heat exposure. The percentage of census-tract area not covered by vegetation (NonVegeRatio) was used to represent limited vegetative cover. The percentage of census-tract area with impervious surfaces (ImpSurfaceRatio) captured the extent of hard, heat-retaining surfaces associated with urban development. The percentage of census-tract area with tree canopy (TreeCanopyRatio) measured local canopy coverage, which may reduce heat exposure through shading and evapotranspiration.

#### 3.2.3 Methodology

This study used a two-stage methodological framework to examine high hypertension burden in a heat-vulnerability context across Maryland census tracts. First, spatial pattern analysis was conducted to describe the geographic distribution of hypertension burden and to identify whether high-burden tracts were spatially concentrated in specific parts of the state. Second, an interpretable machine learning approach combining XGBoost and SHAP was used to predict high hypertension burden and explain the contribution of environmental, demographic, socioeconomic, built-environment, and adaptive-capacity variables.

The spatial pattern analysis was conducted at the census-tract level to describe the geographic heterogeneity of hypertension burden across Maryland. Using the binary high-burden indicator defined in Section 3.2.1, census tracts were grouped into high-burden and lower-burden categories. The high-burden tracts were then mapped to examine whether elevated hypertension burden was randomly distributed or spatially concentrated in particular urban, suburban, or rural areas. This descriptive spatial analysis provided an initial understanding of where hypertension burden was concentrated before applying the predictive model. This binary outcome was also used as the classification target in the subsequent XGBoost model. Because the median-based threshold produced a nearly balanced distribution of high-burden and lower-burden tracts, the outcome was suitable for supervised classification. Mapping the binary outcome further allowed the study to identify visible spatial clustering and compare high-burden areas across Maryland's urban, suburban, and rural contexts.

After describing the spatial distribution of hypertension burden, this study used XGBoost to predict whether each census tract belonged to the high-burden hypertension category. XGBoost was selected because high hypertension burden in a heat-vulnerability context is likely shaped by complex, non-linear, and interactive relationships among heat exposure, land-cover conditions, social vulnerability, demographic susceptibility, baseline health burden, and household cooling access. Compared with conventional linear models, XGBoost is better suited for identifying threshold effects and interaction-dependent patterns in tabular census-tract data (33).

The XGBoost model can be expressed as an additive ensemble of regression trees, as presented in Equation 1:


$$\begin{array}{l} {ŷ}_{i}=\overset{K}{\underset{k=1}{\sum }}f_{k}\left(x_{i}\right),\;f_{k}\mathrm{\in F} \end{array}$$ (1)

where ŷ_*i*_ is the predicted probability or classification score for census tract i, *x*_*i*_ is the vector of predictor variables, *K* is the number of trees, and *f*_*k*_ represents the k-th decision tree.

The objective function minimized by XGBoost is presented in Equation 2:


$$\begin{array}{l} L\;=\;\Sigma_{\left\{i=1\right\}}^{\left\{n\right\}l\left(y_{i},\;y_{i}\right)}+\;\Sigma_{\left\{k=1\right\}}^{\left\{K\right\}\Omega \left(f_{k}\right)} \end{array}$$ (2)

where *l*(*y*_*i*_, *y*_*i*_) is the loss function measuring prediction error and Ω(*f*_*k*_) is the regularization term used to penalize model complexity.

The SHAP explanation model was written as presented in Equation 3:


$$\begin{array}{l} g\left(z^{\prime }\right)=\phi_{0}+\overset{M}{\underset{j=1}{\sum }}\phi_{j}z_{j}^{\prime } \end{array}$$ (3)

Where *g*(*z*′) is the explanation model, phi_0 is the baseline prediction, M is the number of predictors, $z_{j}^{\prime }$ indicates whether predictor j is included in the explanation, and ϕ_*j*_ is the SHAP value representing the contribution of predictor j to the model prediction. SHAP results were interpreted at two levels. Global feature importance was evaluated using the average magnitude of SHAP values to identify which predictors contributed most strongly to model predictions across all census tracts. SHAP summary plots were used to show both the importance and direction of each predictor's effect, while SHAP dependence plots were used to examine non-linear relationships and possible thresholds for key predictors such as older-adult population share, educational disadvantage, summer air temperature, and lack of air conditioning.

For model development, the full census-tract dataset was divided into training and testing subsets using a 70/30 stratified train-test split. Stratification was used to preserve the proportion of high-burden and lower-burden census tracts in both subsets. Hyperparameter tuning was conducted within the training set using five-fold cross-validation, and the final XGBoost model was evaluated on the independent 30% test set. A fixed random seed of 42 was used to ensure reproducibility. To reduce direct spatial leakage, census-tract identifiers, geographic coordinates, spatial lag variables, and neighboring outcome values were not included as model predictors. However, because nearby census tracts may share similar socioeconomic, environmental, and built-environment characteristics, random splitting cannot fully eliminate spatial dependence. Therefore, the model performance should be interpreted as predictive performance within the Maryland census-tract context rather than as evidence of spatially independent generalization.

### 3.3 Analytic framework

**Figure 2:**
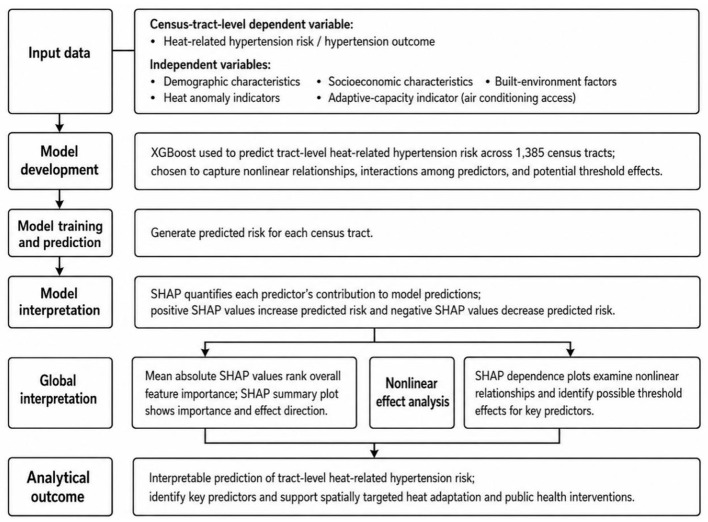
Analytic framework linking input variables, binary hypertension-burden classification, XGBoost prediction, model evaluation, and SHAP interpretation.

The analytic framework, as shown in Figure 2, begins with census-tract-level input data that include the dependent variable of high hypertension burden in a heat-vulnerability context and a set of independent variables representing demographic characteristics, socioeconomic conditions, built-environment factors, heat anomaly indicators, and adaptive capacity. These variables are used to develop an XGBoost model to predict tract-level high hypertension burden in a heat-vulnerability context across 1,385 census tracts. XGBoost is used because it can capture non-linear relationships, interactions among predictors, and potential threshold effects that may not be adequately represented by conventional linear models.

After model training and prediction, SHAP is applied to interpret the XGBoost results. SHAP values quantify the contribution of each predictor to the predicted risk for each census tract, with positive values indicating an increase in predicted risk and negative values indicating a decrease. The interpretation proceeds in two complementary directions: global interpretation, using mean absolute SHAP values and SHAP summary plots to identify the most influential predictors and their effect directions; and non-linear effect analysis, using SHAP dependence plots to examine non-linear relationships and possible thresholds among key predictors.

## 4 Results

### 4.1 Descriptive statistics & spatial heterogeneity of heat-health risk

Table 2 presents descriptive statistics for the dependent variable and all predictors used in the analysis. Across the 1,385 Maryland census tracts, 695 tracts, or approximately 50.2%, are classified as high-burden hypertension tracts, while 690 tracts are classified as lower-burden tracts. This nearly balanced distribution provides a suitable basis for modeling spatial variation in high hypertension burden in a heat-vulnerability context.

**Table 2:** Descriptive statistics of model variables across MD census tracts (*N* = 1,385).

Variable	Mean	SD	Min	Max
High hypertension burden (binary)	0.502	0.5	0	1
Population aged over 65 (%)	12.705	6.927	0.2	89
African-American population (%)	31.129	30.7	0	99.355
Hispanic population (%)	9.286	12.008	0	93.769
Population below poverty line (%)	10.552	9.676	0	87.984
Gini index of income inequality	0.402	0.062	0	0.703
Population with no health insurance (%)	60.78	6.245	0	98.866
Population with less than high school diploma (%)	10.682	8.117	0.137	77.525
Population living alone (%)	14.618	6.666	0	47.584
Householders over 65 living alone (%)	32.332	15.678	0	95.293
Households without air conditioning (%)	1.17	0.291	0	2.353
Census-tract area not covered by vegetation (%)	69.271	28.786	6.208	100
Census-tract area with impervious surfaces (%)	34.308	16.161	8.017	93.753
Census-tract area with tree canopy (%)	27.272	16.55	0	73.503
Summer land surface maximum temperature (°F)	79.212	2.177	73.837	92.084
Summer maximum air temperature (°F)	84.104	2.365	77.8	87.7

Percentage-based variables are reported in percentage units. High hypertension burden is a binary indicator, where 1 represents census tracts classified as having high hypertension burden and 0 represents lower-burden tracts.

The share of residents aged over 65 ranges from nearly 0% to 89.0%, while the proportion of African-American residents ranges from 0% to 99.4%. Socioeconomic indicators also show wide variation: the poverty rate ranges from 0% to 88.0%, the share of residents with less than a high school diploma ranges from nearly 0% to 77.5%, and the percentage of householders over 65 living alone reaches as high as 95.3% in some tracts. Built-environment and heat-exposure variables similarly vary across the state. The percentage of land not covered by vegetation ranges from 6.2% to 100%, impervious surface coverage ranges from 8.0% to 93.8%, summer land surface maximum temperature ranges from approximately 74.0°F to 92.1°F, and summer maximum air temperature ranges from 77.8°F to 87.7°F. These ranges suggest that Maryland census tracts differ sharply in both population vulnerability and environmental heat exposure.

Spatially, high-burden hypertension tracts are not evenly distributed across Maryland. They are concentrated in several urban, suburban, and rural clusters, including Baltimore City, Prince George's County, parts of southern Maryland, western Maryland, and portions of the Eastern Shore. Counties such as Kent, Garrett, Washington, Allegany, Worcester, Dorchester, Wicomico, Baltimore City, and Prince George's show relatively high proportions of high-burden tracts, whereas Howard, Montgomery, Frederick, Calvert, Carroll, and Anne Arundel contain larger shares of lower-burden tracts. This pattern indicates that hypertension burden is spatially heterogeneous at the census-tract level and cannot be adequately represented by statewide averages or county-level summaries alone.

**Figure 3:**
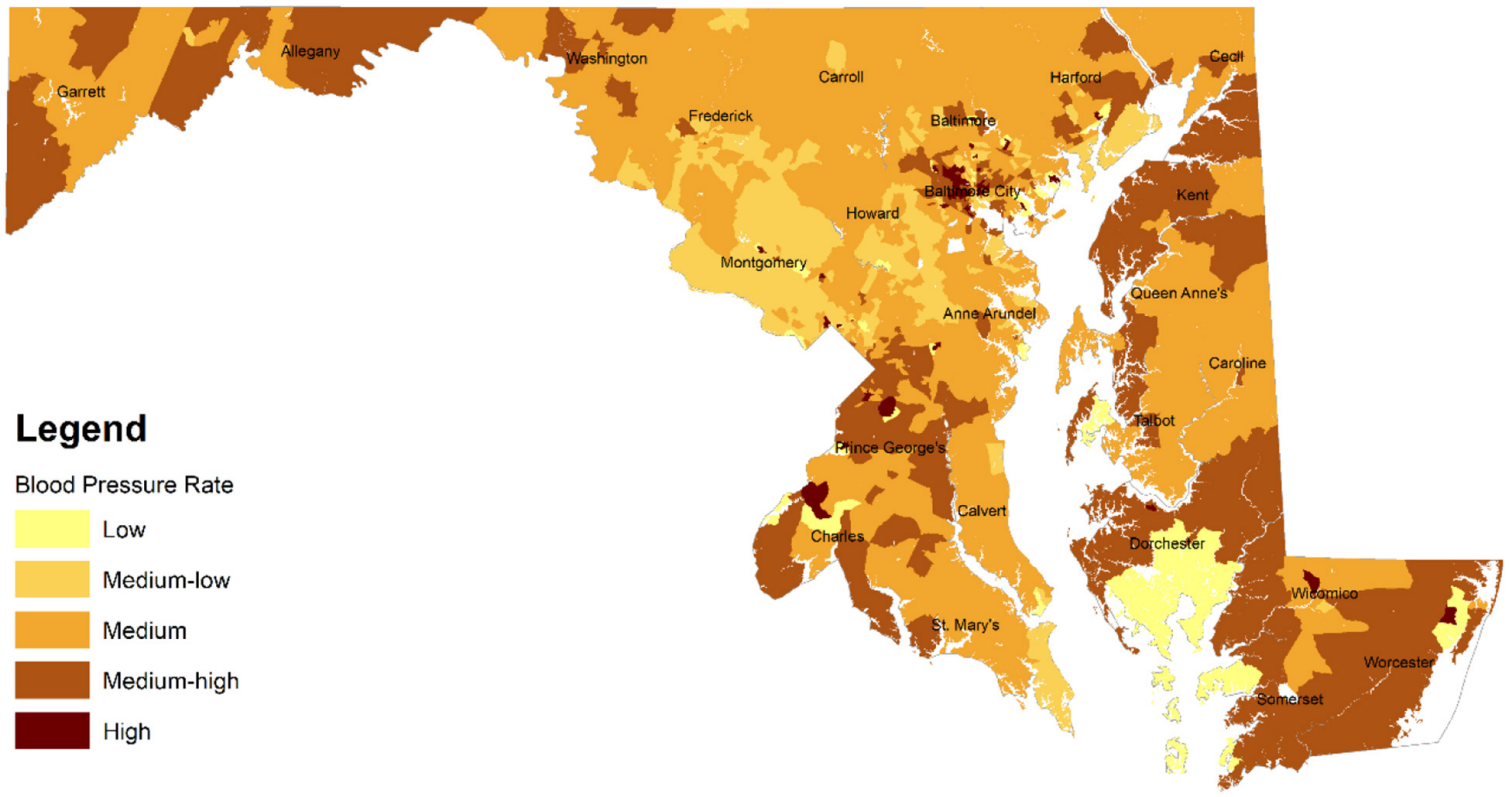
Spatial distribution of hypertension burden across Maryland census tracts.

Figure 3 shows substantial spatial heterogeneity in Marylanders' hypertension burden. Higher hypertension prevalence is not randomly distributed, but concentrated in several distinct clusters. The most visible high-burden areas appear in and around Baltimore City, parts of Prince George's County, Charles County, southern Maryland, and portions of the Eastern Shore, including Dorchester, Somerset, Wicomico, Worcester, and Kent Counties. Several census tracts in these areas fall into the medium-high or high categories, indicating localized concentrations of elevated hypertension burden.

By contrast, lower hypertension burden is more evident in parts of central Maryland, especially portions of Montgomery, Howard, Anne Arundel, and Frederick Counties, where many census tracts fall into the low or medium-low categories. Some lower-burden tracts are also visible along parts of the Chesapeake Bay and in selected coastal or suburban areas. This pattern suggests that hypertension burden varies sharply even within the same county, highlighting the importance of census-tract-level analysis rather than relying only on county-level averages.

### 4.2 XGBoost model performance

The XGBoost model demonstrated strong overall predictive performance in classifying census tracts with elevated high hypertension burden in a heat-vulnerability context, as shown in Table 3. The model achieved an accuracy of 0.819 and a balanced accuracy of 0.806, indicating that it performed well across both high-risk and lower-risk classes rather than being driven only by the majority class. The ROC-AUC value of 0.821 further suggests good discriminatory ability, meaning that the model was effective in distinguishing census tracts with higher predicted high hypertension burden in a heat-vulnerability context from those with lower risk.

**Table 3:** Model performance metrics.

Metric	Value
Accuracy	0.819
Balanced accuracy	0.806
Precision	0.914
Recall	0.833
F1-score	0.872
ROC-AUC	0.821
PR-AUC	0.909

The model also showed particularly high precision, with a value of 0.914. This indicates that census tracts classified by the model as high risk were very likely to be true high-risk tracts. The recall value of 0.833 suggests that the model successfully identified most high-risk tracts, although a smaller portion of high-risk areas may still have been missed. The resulting F1-score of 0.872 reflects a strong balance between precision and recall. In addition, the PR-AUC value of 0.909 indicates robust performance in identifying high-risk tracts, which is especially important for public health applications where accurately detecting vulnerable areas is a priority.

**Figure 4:**
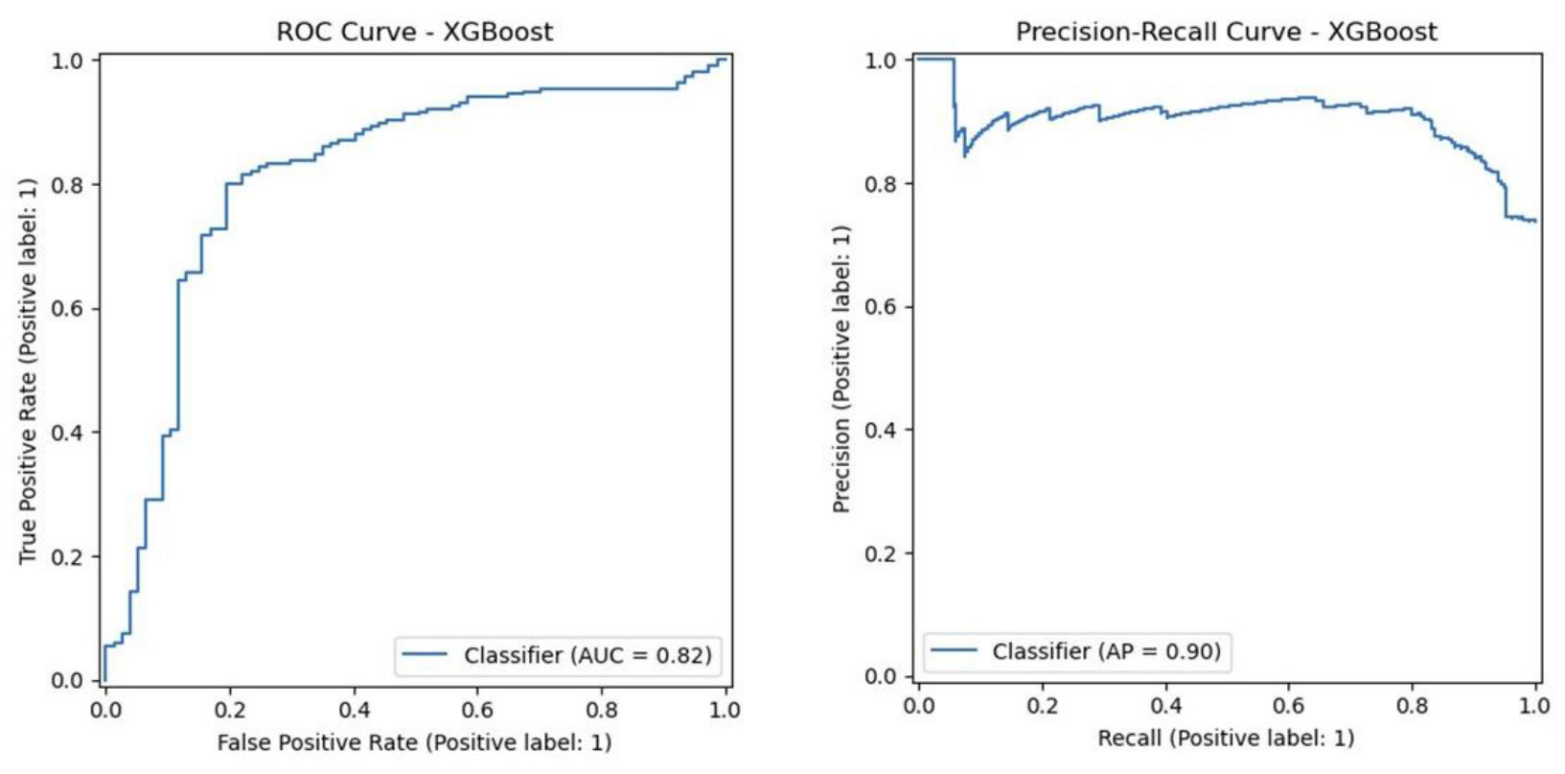
ROC and precision-recall curves.

Figure 4 showcased two key metrics of XGBoost model performance. The XGBoost model achieved an ROC-AUC of 0.82, indicating good discriminatory ability in distinguishing census tracts with high hypertension prevalence from those with lower hypertension prevalence. The model also achieved an average precision of 0.90, suggesting strong performance in identifying high-prevalence hypertension tracts while maintaining a relatively low false-positive rate. These two metrics showed good classification performance for identifying high-prevalence tracts.

### 4.3 Global SHAP feature importance

**Figure 5:**
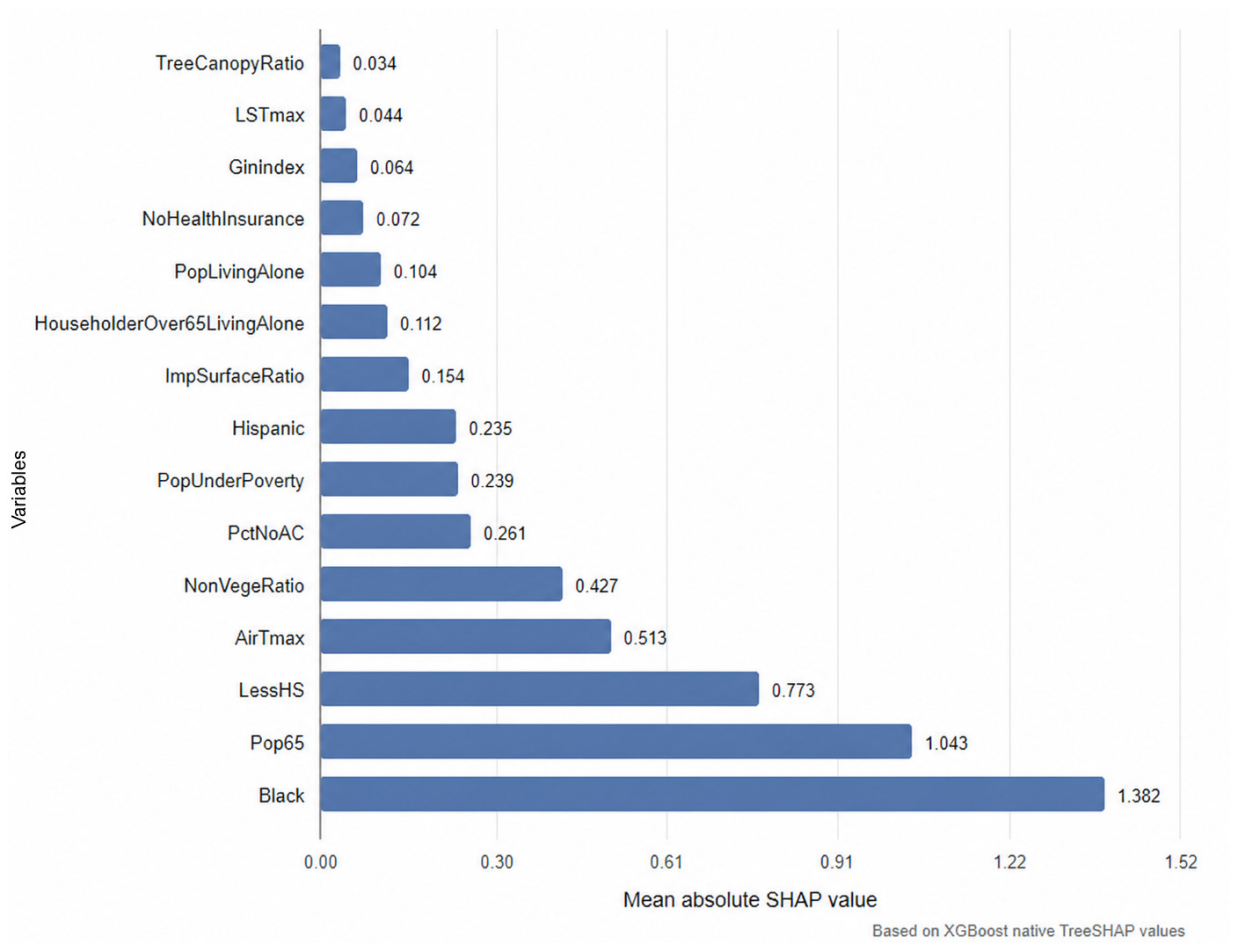
Global SHAP feature importance ranking.

Figure 5 presents the global SHAP feature importance ranking for the XGBoost model predicting census tracts with high hypertension burden. The ranking is based on the mean absolute SHAP value, which measures the average magnitude of each variable's contribution to model predictions. The results show that the percent population as African-American only was the most influential predictor, with a mean absolute SHAP value of 1.382. This was followed by the percent population aged over 65, with a value of 1.043, and the percent population with less than a high school diploma, with a value of 0.773. These three variables contributed substantially more than the remaining predictors, indicating that racial composition, age structure, and educational disadvantage were the dominant factors used by the model to distinguish census tracts with high blood pressure prevalence.

Heat-related and land-cover variables formed the second tier of predictors. Air temperature anomaly in summer ranked fourth, with a mean absolute SHAP value of 0.513, followed by the percent census-tract area not covered in vegetation, with a value of 0.427. This suggests that summer air temperature conditions and limited vegetation coverage provided additional predictive information beyond demographic and socioeconomic characteristics. Adaptive capacity and economic disadvantage variables had moderate contributions, including the percent households with no air conditioning, with a value of 0.261; the percent population below the poverty line, with a value of 0.239; and the percent population as Hispanic, with a value of 0.235. The percent census-tract urban area with impervious surfaces contributed less strongly, with a mean absolute SHAP value of 0.154.

Several variables had comparatively limited influence on the model output. The percent householder over 65 living alone, percent population living alone, percent population with no health insurance, Gini index of income inequity, land surface heat anomaly in summer, and percent census-tract area with tree canopy all had relatively small mean absolute SHAP values. In particular, land surface heat anomaly in summer (LSTmax) and tree canopy coverage contributed little to the model after accounting for the stronger demographic, socioeconomic, and summer air-temperature predictors.

**Figure 6:**
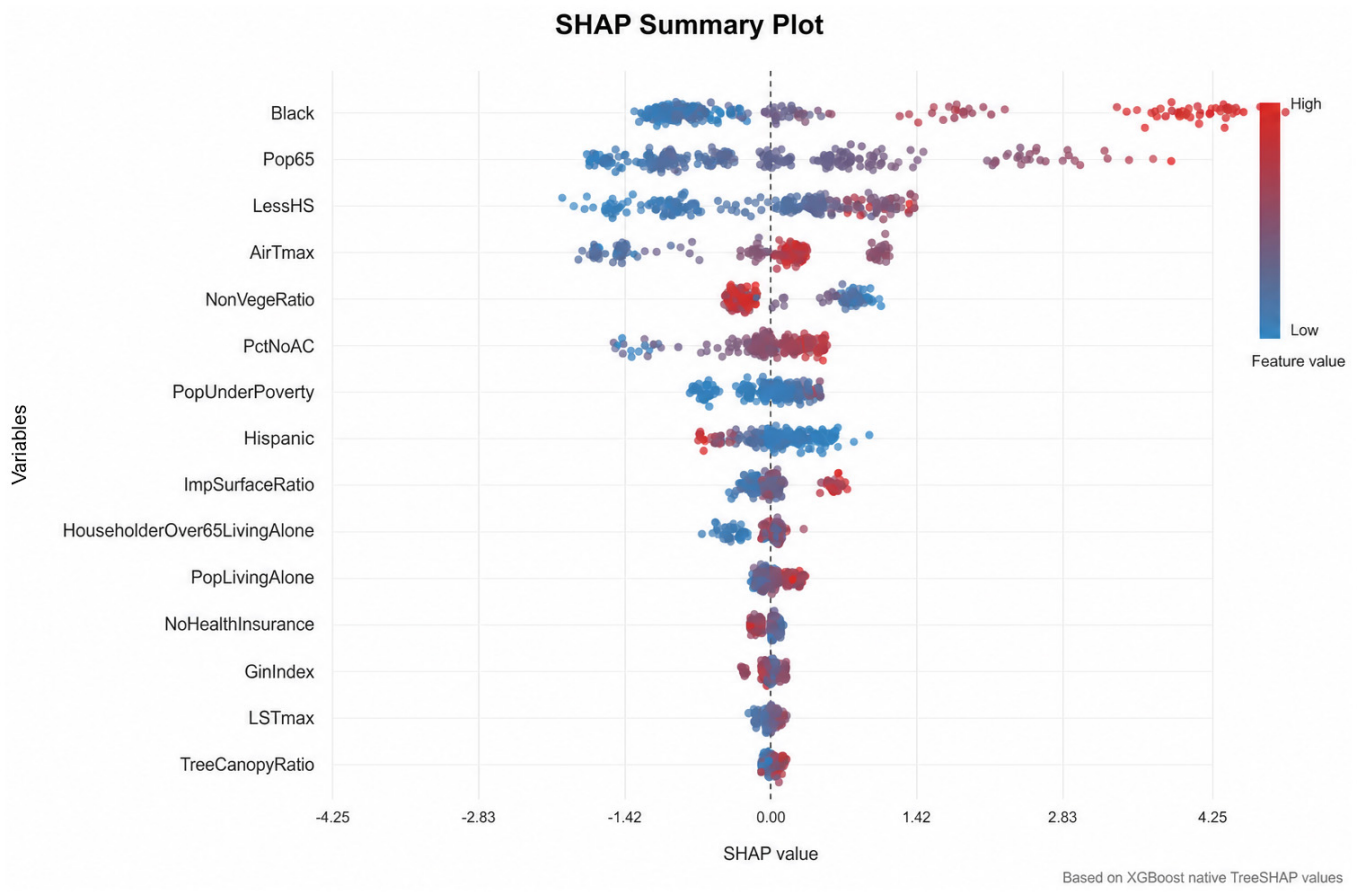
SHAP summary plot for predicting high hypertension burden.

This study complements the global importance ranking by showing the direction and distribution of SHAP effects across census tracts, as shown in Figure 6. For the percent population as African-American only (Black), high feature values are concentrated on the positive side of the SHAP axis, while low values are concentrated on the negative side. This indicates that census tracts with higher African-American population shares were more likely to be classified by the model as areas with high blood pressure prevalence. However, it's worth noticing that this variable is measured at the census-tract level and should be understood as a proxy for structural and spatial inequality, including residential segregation, unequal neighborhood resources, differential environmental exposure, housing conditions, and health-care access. It should not be interpreted as an individual-level causal factor or as evidence of any biological relationship between race and hypertension burden. A similar directional pattern is observed for the percent population aged over 65 (Pop65): higher proportions of older adults generally increased the predicted probability of high blood pressure prevalence.

Figure 6 also shows that the effects of several predictors were heterogeneous across census tracts. For the percent population with less than a high school diploma (LessHS), higher values generally shifted predictions toward the high-prevalence class, but the spread of SHAP values suggests that the magnitude of this effect varied across observations. Air temperature anomaly in summer (AirTmax) showed a clear positive contribution for higher values, indicating that census tracts with higher summer air temperature anomalies tended to receive higher predicted probabilities of high blood pressure prevalence. The percent census-tract area not covered in vegetation (VegeRatio) also displayed a positive contribution, suggesting that less vegetated areas were associated with model predictions of higher blood pressure prevalence.

In contrast, lower-ranked variables in Figure 6 had SHAP values clustered close to zero, indicating limited directional influence. Variables such as the Gini index of income inequity (GiniIndex), land surface heat anomaly in summer (LSTmax), and percent census-tract area with tree canopy (TreeCanopyRatio) showed narrow SHAP distributions, suggesting that they played a minor role in shifting individual tract-level predictions.

### 4.4 Non-linear effects of key predictors

**Figure 7:**
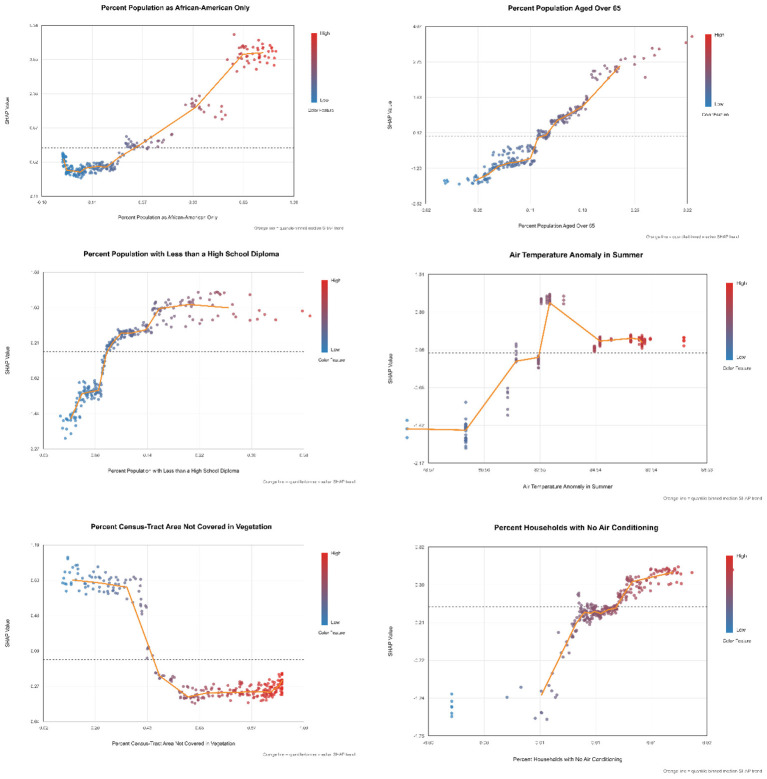
SHAP dependence plot top 6 influencing variables.

Figure 7 illustrates the non-linear marginal influence of the key predictors on model-predicted high hypertension burden in a heat-vulnerability context, using SHAP dependence plots. In these plots, positive SHAP values indicate that a given predictor increases the predicted risk relative to the model baseline, whereas negative SHAP values indicate a risk-reducing contribution. The orange trend line summarizes the quantile-binned median SHAP response and therefore highlights the dominant non-linear pattern for each variable.

The percentage of the population identified as African-American shows a strongly non-linear positive association with predicted high hypertension burden in a heat-vulnerability context. At relatively low values, this variable contributes negatively or only weakly to the predicted outcome. However, the SHAP response increases rapidly once the proportion rises above approximately 0.30–0.40, with particularly large positive contributions at the highest values. This pattern suggests that the model identifies areas with higher African-American population shares as having substantially elevated predicted risk, likely reflecting broader spatial patterns of structural disadvantage, differential heat exposure, health-care access, housing conditions, and cumulative socioeconomic vulnerability rather than any biological interpretation of race.

The proportion of residents aged over 65 also exhibits a monotonic but non-linear relationship with predicted risk. Low values of this predictor are associated with negative SHAP contributions, while the effect becomes increasingly positive as the elderly population share rises. The increase is especially pronounced beyond roughly 0.12–0.15, indicating a threshold-like response in which communities with larger elderly populations experience disproportionately higher predicted high hypertension burden in a heat-vulnerability context. This is consistent with the physiological vulnerability of older adults to heat stress and the greater prevalence of chronic cardiovascular conditions in aging populations.

Educational disadvantage, measured as the percentage of the population with less than a high school diploma, displays a steep positive response at relatively low-to-moderate values. The SHAP trend shifts from negative to positive as the share of low educational attainment increases, then rises sharply before reaching a plateau at higher values. This suggests that increases in educational deprivation initially have a strong marginal effect on predicted risk, but the effect diminishes once disadvantage becomes widespread. The plateau may indicate saturation, where additional increases in low educational attainment provide less incremental information because the model has already classified these areas as highly vulnerable.

Summer maximum air temperature shows a distinct threshold-type relationship. Lower temperature values are associated with negative SHAP contributions, indicating reduced predicted high hypertension burden in a heat-vulnerability context. As temperature increases into the low-to-mid 80s, the SHAP values rise sharply and become positive, suggesting that the model detects a critical range at which heat exposure begins to substantially elevate predicted risk. At higher temperatures, the effect remains positive but appears to stabilize or slightly decline, implying a non-linear exposure-response relationship rather than a simple linear increase. This may reflect interactions with adaptation, spatial confounding, or the influence of other heat-related and socioeconomic predictors in the model.

The percentage of census-tract area not covered by vegetation presents a more complex and counterintuitive non-linear pattern. Lower values of non-vegetated area are associated with positive SHAP contributions, while intermediate-to-high values are associated with negative or weakly negative contributions. This indicates that, conditional on the other predictors in the model, greater non-vegetated land cover does not uniformly increase predicted risk. The pattern may reflect interaction effects, differences between dense urban, suburban, and rural land-use contexts, or collinearity with other environmental and socioeconomic variables. Therefore, this variable should be interpreted as a conditional model effect rather than a direct causal estimate of vegetation loss.

The percentage of households without air conditioning shows a clear positive non-linear effect. At the lowest values, the SHAP contribution is negative, but the effect rises sharply as the proportion of households without air conditioning increases. Beyond a relatively narrow threshold, the contribution becomes positive and continues to increase, indicating that lack of air-conditioning access is an important amplifier of predicted high hypertension burden in a heat-vulnerability context. This relationship is consistent with the role of residential cooling access as a protective adaptation mechanism during extreme heat events.

## 5 Discussion & limitations

This study used an interpretable machine learning framework to examine tract-level variation in high hypertension burden in a heat-vulnerability context across Maryland. The results show that the XGBoost model performed well in distinguishing census tracts with high hypertension burden from those with lower burden, indicating that the selected environmental, demographic, socioeconomic, built-environment, and adaptive-capacity variables contain meaningful information for predicting spatial differences in hypertension risk. More importantly, the SHAP results reveal that the model predictions were driven primarily by social and demographic factors, while heat exposure and adaptive-capacity variables provided additional but secondary explanatory value.

One of the most important findings is that the percentage of the population identified as African-American was the strongest predictor in the model. This result should not be interpreted as evidence of any biological relationship between race and hypertension risk. Rather, it likely reflects the cumulative effects of structural inequality, residential segregation, unequal access to health-promoting resources, differential environmental exposure, housing conditions, and health-care access. This finding is consistent with environmental justice research showing that racialized communities often experience disproportionate exposure to environmental hazards and fewer adaptive resources. In the context of hypertension burden under heat vulnerability, the result suggests that race-related spatial patterns may operate as a proxy for broader structural and neighborhood-level inequities.

The strong contribution of older-adult population share is consistent with existing heat-health research. Older adults are generally more physiologically sensitive to heat because of reduced thermoregulatory capacity, higher prevalence of chronic disease, medication use, and greater likelihood of mobility limitations or social isolation. The SHAP dependence results further suggest that the association is non-linear: the predicted risk increases more sharply once the proportion of older adults exceeds a moderate level. This threshold-like pattern indicates that age-related vulnerability may become particularly important in census tracts where older residents are spatially concentrated.

Educational disadvantage was also a dominant predictor of high hypertension burden. The non-linear pattern suggests that increases in the percentage of residents with less than a high school diploma initially raise predicted risk substantially, but the effect appears to plateau at higher levels. This may indicate that education functions as an early marker of neighborhood socioeconomic vulnerability, health literacy, employment opportunity, and access to preventive health resources. Once a tract is already characterized by substantial educational disadvantage, additional increases may provide less incremental information to the model because the area has already been identified as highly vulnerable.

A notable finding is that heat-related variables were important, but they were not the strongest predictors. Summer maximum air temperature contributed meaningfully to model predictions and showed a threshold-type relationship with predicted risk, but demographic and socioeconomic variables ranked higher. This suggests that high hypertension burden in a heat-vulnerability context is not simply determined by where temperatures are highest. Instead, heat appears to amplify pre-existing social and health vulnerabilities. In other words, the health burden of heat is shaped by the interaction between exposure and susceptibility, rather than exposure alone.

Another important and somewhat unexpected result concerns the built-environment variables. Non-vegetated land area contributed to the model, but its dependence pattern was not uniformly positive. In addition, tree canopy and land surface temperature had relatively limited contributions compared with demographic, socioeconomic, and air-temperature variables. This differs from many heat vulnerability studies that emphasize vegetation, impervious surfaces, and land surface temperature as central determinants of heat risk. One possible explanation is that the effect of vegetation and land-cover conditions may depend on local context. For example, non-vegetated land may represent dense urban development in some tracts, but agricultural land, open space, or other non-urban surfaces in others. Similarly, land surface temperature may overlap with air temperature, urbanization, or vegetation indicators, reducing its independent contribution in the model. These findings suggest that built-environment variables should be interpreted conditionally rather than as universally direct predictors of hypertension risk.

The role of air-conditioning access is more consistent with prior heat-health research. The percentage of households without air conditioning showed a positive non-linear association with predicted risk, indicating that limited cooling access can amplify high hypertension burden in a heat-vulnerability context. Although the overall variation in lack of air conditioning across Maryland census tracts is relatively small, the SHAP results suggest that even modest differences in cooling access may matter when combined with social vulnerability, chronic disease burden, and elevated heat exposure. This finding supports the view that air conditioning should be treated not only as a household amenity, but also as an adaptive-capacity indicator relevant to heat-health planning.

Several directions can further strengthen this line of research. First, this study uses CDC PLACES small-area estimates of high blood pressure prevalence, which are currently among the most spatially detailed and publicly available population health indicators for census-tract analysis. Future studies could integrate clinical records, insurance claims, or longitudinal cohort data to validate tract-level estimates and better distinguish estimated hypertension risk from individually observed disease occurrence. Second, the present cross-sectional design is appropriate for identifying spatially patterned predictors but cannot establish causal relationships. Future work could incorporate longitudinal exposure histories, repeated heat-event observations, or quasi-experimental designs to examine whether changes in heat exposure, greening, housing conditions, or cooling access are followed by changes in hypertension-related outcomes. Third, several predictors in this study may reflect overlapping socio-environmental processes. Heat exposure, impervious surfaces, vegetation, and urbanization are interrelated, just as race, poverty, education, health-care access, and housing conditions jointly capture structural vulnerability. Future research could combine interpretable machine learning with causal inference, spatial econometric models, or multilevel designs to better separate these mechanisms. Fourth, heat exposure is dynamic, whereas this study relies on tract-level summary indicators constrained by available data sources, spatial resolution, and aggregation procedures. Future work could add daily heat-event metrics, indoor temperature estimates, personal exposure data, or time-activity information to capture more precise exposure pathways. Fifth, although Maryland provides a useful case for examining heat-health vulnerability across heterogeneous urban, suburban, and rural contexts, comparative applications in other states or metropolitan regions would help assess whether similar predictors, non-linear thresholds, and equity patterns emerge under different climatic, demographic, and health-care conditions. Finally, although census-tract identifiers, geographic coordinates, spatial lag variables, and neighboring outcome values were excluded from the model to reduce direct spatial leakage, the use of a random train-test split may not fully eliminate spatial dependence among nearby census tracts. Future studies could use spatial cross-validation or geographically blocked validation to further test the model's generalizability across spatial contexts.

## 6 Conclusions & policy implications

This study examined high hypertension burden in a heat-vulnerability context across 1,385 census tracts in Maryland using an interpretable machine learning framework. By combining XGBoost with SHAP, the analysis provided both accurate tract-level prediction and transparent interpretation of the factors driving model outputs. The model demonstrated strong performance in identifying census tracts with high hypertension burden, supporting the use of machine learning for fine-scale heat-health risk assessment.

The findings show that high hypertension burden in a heat-vulnerability context in Maryland is spatially heterogeneous and shaped by more than heat exposure alone. The most influential predictors were African-American population share, older-adult population share, and low educational attainment, followed by summer maximum air temperature, non-vegetated land area, and lack of air conditioning. These results indicate that the burden of heat-related hypertension is closely tied to structural social vulnerability, demographic susceptibility, environmental exposure, and household adaptive capacity.

This study contributes to the heat-health literature in three main ways. First, it focuses on hypertension as a specific cardiovascular outcome, extending prior research that has often relied on general heat vulnerability, mortality, or hospitalization measures. Second, it demonstrates the usefulness of interpretable machine learning for detecting non-linear and threshold-like relationships among heat-health predictors. Third, it provides spatially explicit evidence that can support targeted public health planning, cooling assistance, heat preparedness, and neighborhood-scale adaptation strategies.

These findings provide practical implications for spatially targeted and equity-oriented heat-health planning. The results suggest that local adaptation strategies should not rely on heat exposure alone, because high hypertension burden in a heat-vulnerability context is shaped by the overlap of demographic susceptibility, socioeconomic disadvantage, environmental exposure, and adaptive capacity. Public health agencies, emergency management offices, and planning departments can use the SHAP results to identify census tracts where multiple risk factors converge, such as high older-adult concentration, low educational attainment, elevated summer air temperature, limited vegetation, and low air-conditioning access. These areas should be prioritized for integrated interventions, including cooling assistance, targeted heat-warning communication, wellness checks for older residents, expanded access to cooling centers, and neighborhood-scale greening or heat-mitigation investments. More broadly, reducing hypertension burden under heat vulnerability requires not only lowering thermal exposure, but also addressing the social and infrastructural inequalities that limit residents' capacity to prepare for, avoid, and recover from extreme heat.

## Data Availability

The original contributions presented in the study are included in the article/supplementary material, further inquiries can be directed to the corresponding author.
